# Development of the Arterial Supply of the Spinal Cord Tissue Based on Radioanatomical and Histological Studies in Cattle

**DOI:** 10.1007/s00062-021-01093-3

**Published:** 2021-09-28

**Authors:** Armin Thron, Peter Stoeter, Jasmin Schiessl, Andreas Prescher

**Affiliations:** 1 Clinic for Diagnostic and Interventional Neuroradiology, University Hospital RWTH Aachen, Pauwelsstr. 30, 52057 Aachen, Germany; 2 Theaterstr. 31, 52062 Aachen, Germany; 3 Department of Radiology, CEDIMAT, Plaza de la Salud, Santo Domingo, Dominican Republic; 4 Friedrich-Ebert-Straße 70, 04109 Leipzig, Germany; 5 Institute of Molecular and Cellular Anatomy, University Hospital RWTH Aachen, Prosektur, Wendlingweg 2, 52074 Aachen, Germany

**Keywords:** Anterior spinal artery, Burst of vascularization, Embryology, Microangiography, Spinal cord blood supply

## Abstract

**Purpose:**

Angiographic techniques have gained increasing importance in suspected vascular disease of the spinal cord. This demands an advanced understanding of spinal cord blood vessel anatomy and its embryologically founded broad spectrum of variations. The aim of this study was to improve knowledge on contentious issues concerning the development of spinal cord arterial supply in higher mammals and to offer visual information of high didactic value.

**Methods:**

The prenatal development was examined in cattle, using multiplanar high-resolution microangiography of injected specimens and microscopic sections. The gestational ages of the 15 specimens were between the late embryonic and the early fetal period (5–11 weeks). Microangiography of the human spinal cord from an earlier published study were used to envisage an adult arterial vascularization pattern in higher mammals.

**Results:**

Establishment of the unpaired anterior spinal artery (ASA) goes through two procedures of reconfiguration until achieving its final design. Regression of the primarily established anteromedian tract is observed in cattle fetuses of 9–10 weeks. Return to the ontogenetic disposition of bilateral symmetry and a burst of vascularization from all parts of the spinal meninges follow and include the anterior median fissure as a preferred vascular pathway. Large sulcal/central arteries longitudinally anastomosing between each other emerge on both sides of the midline. The embryological pattern of exclusive peripheral medullary supply must have been converted into a combined system of predominant central (centrifugal) supply of the enlargements before a final unpaired ASA can be reconstructed.

**Conclusion:**

Previous investigators focused on the early embryonic development of spinal cord arteries and missed the profound remodeling of the vascular architecture in the early fetal period.

## Introduction

Contrary to detailed and systematic studies on the development of the cranial arteries in humans [1–3], investigations on embryonic and/or fetal development of the spinal cord blood vessels in humans are rare and less comprehensive [4, 5]. For at least a century, individual case observations in human embryos [5–7] together with findings from comparative studies in animals have indicated a high degree of similarity in this development among higher vertebrates [8–10]. Observations gained from cattle should therefore not essentially differ from the prenatal development of spinal cord arteries in humans.

In 1976 a post-mortem radiological technique of prenatal microangiography was reported [11], which was employed to study the development of blood vessels in different parts of animal brains, mainly cattle [12–14]. The main advantage of this post-mortem X‑ray technique, first described in 1911 [15] more than a century ago, is the possibility it offers to evaluate the vasculature in differently oriented sections of specimens following radio-opaque injections. Microangiograms obtained from thin slices of the object avoid excessive superimposition effects and provide images of blood vessels in high-resolution down to diameters between 20 µ and 5 µ [16]. This technique also allows separate visualization of arteries or veins, depending on the injection site and the chosen properties of the contrast medium. These significant advantages were again confirmed in a detailed post-mortem study on the vascular anatomy of the human spinal cord [17, 18].

The vascular anatomy of the spinal cord is well-known for its broad spectrum of anomalies within the range of normal variation; however, summarizing embryologically defined vascular variations was not the primary goal of this research. The application of these unique imaging techniques aimed at clear anatomical illustrations reflecting the nature and perhaps the mechanisms of vascular reorganization processes like the formation of an unpaired anterior spinal artery or changing intrinsic supply patterns. In the first half of the last century, the early embryonic period of medullary blood supply development was studied in detail [6, 8–10, 19, 20]; however, knowledge concerning the late embryonic and early fetal interval remained limited. This gap of knowledge affects a developmental period, in which circulatory adaptations become necessary to fulfil the metabolic needs of the maturating spinal cord. Consequently, changes that follow the primitive design of intrinsic vascularization have not received much attention in the spinal cord. The same applies to the impact of the development of the anterior median fissure, a deep infolding of a duplication of meninx primitiva as a result of expanding anterior horns. The number of cases in this study are not large enough to provide statistical results but the techniques applied here are supposed to contribute to scientific anatomical evaluations and to offer images of high value for teaching purposes.

## Material and Methods

For the radioanatomical and histological investigations, 15 cattle embryos and fetuses were evaluated in each group. The animal specimens were found in slaughtered cattle and kept for research. The vertex-breech length (VBL) of the embryos and early fetuses varied from 20 mm to 130 mm, corresponding to gestational ages (GA) of 5–11 weeks [21]. In three specimens, the spinal cord was examined in situ together with the vertebral column. The study also included one example of segmental supply in a chick embryo. This was part of a pilot study comprising three chick embryos examined at 6 days of incubation in order to obtain information about the very early development of perineural vascular plexuses and segmental supply in vertebrates. Histology was obtained from a different recent series of 14 cattle specimens of corresponding GA. Material for evaluation was taken from the cervical, thoracic and lumbar regions of each spinal cord and stained with hematoxylin and eosin (H&E).

**Fig. 1:**
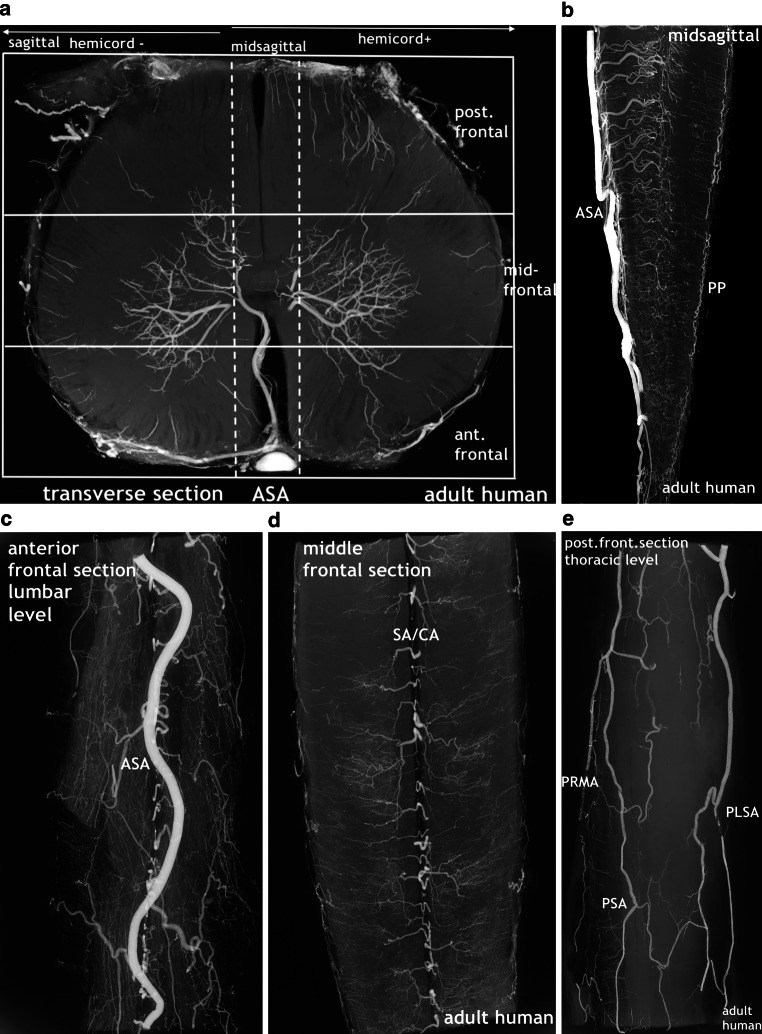
Orientation of the sections cut for the microangiographic evaluation, exemplified with adult human spinal cord specimens. **a** The transverse section at lumbar level indicates the position of the two additional planes in which sections were obtained in this study in order to obtain a three-dimensional perspective on the specimen. Sagittal sections (**b**) may be exclusive midline sections or they may consist of the two halves of a cord segment, one including the midline blood vessels (hemicord+). Frontal sections (**c**–**e**) comprise sections of the anterior and posterior surface and a section in between. Slice thickness varies from 1–3 mm. To image microangiography using spectroscopic plates, the length of frontal and sagittal sections was restricted to a maximum of 2.5 cm. Anterior spinal artery (ASA); sulcal/central artery (SA/CA); posterior radiculomedullary artery (PRMA); posterolateral spinal artery (PLSA); posterior spinal artery (PSA) (**a**–**d** Reproduced from [17])

To accomplish post-mortem angiography of the prenatal material, the fetal specimens were filled with a solution of barium sulphate suspension through arteries of the umbilical cord. The progression of the injected solution into venous channels was critically checked. The abbreviation A/V was used in the image caption when in cases of concomitant arteries and veins a strictly arterial filling of the primitive vasculature was inconclusive. Before fixation, a radiograph of the entire cord was taken using a soft tissue technique. Following preservation, the specimens were cut with microtome blades into 1–3 mm horizontal, frontal or sagittal sections from which high-resolution contact radiographs could be obtained, as exemplified in (Fig. 1). This was done following Paraplast embedment using a special microstructure X‑ray device. The preparation of the contrast medium, injection techniques and microradiographic technique using Kodak spectroscopic plates have been described in detail [3, 11–14, 17, 18]. To avoid problems arising from divergent nomenclatures and in order to maintain consistency of different anatomical entities, we restricted the use of the term tract to the longitudinally extended compaction of capillaries constituting the perineural vascular plexus and for the primordial vascular tissue layers of the primitive meninges. The term primitive anterior spinal artery (pASA) was used to describe this artery from its appearance as a defined vascular structure anteriorly, be it single or double (D-pASA), until its regression in the early fetal period. The adult human microangiograms were selected from an investigation prepublished in textbooks.

## Observations (Results)

### 6-Day Chick Embryo (Fig. 2)

**Fig. 2:**
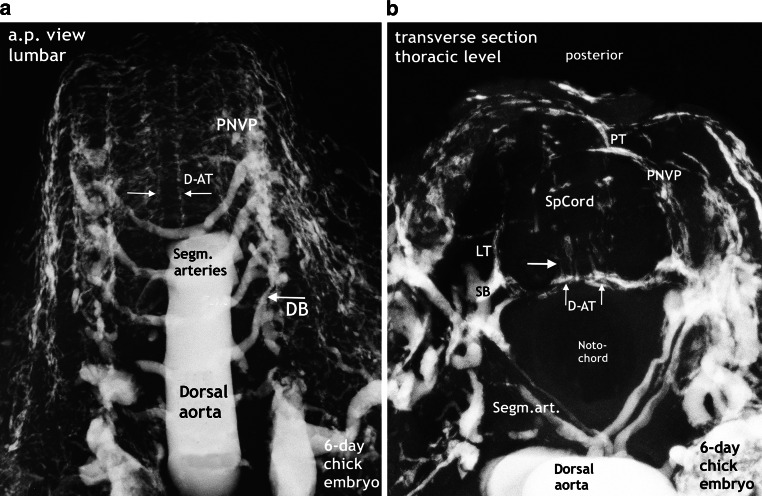
Metameric supply in chick. Segmental arteries, perineural vascular plexus (*PNVP*), doubled anterior tracts (*D‑AT*), and intrinsic capillaries. **a** Microradiogaph of an injected 6‑day chick embryo. Paired segmental arteries originate from the dorsal aorta. Vertebromedullary supply is provided by the dorsal branch (*DB*) of segmental arteries (arrow). The PNVP, which covers the anterior surface of the cord, develops doubled longitudinal anastomotic tracts (D-AT). **b** Same specimen. The oblique craniocaudal view on a transverse section illustrates the position of the notochord and the neural canal enclosing the spinal cord (*SpCord*). Vascular supply can be followed from the dorsal branch of the segmental artery to the spinal branch (*SB*) and to the surface of the cord. The cord is covered by a network of perineural capillaries, regionally thickened as primitive lateral, posterior and doubled anterior tracts (*LT, PT, D‑AT*, *vertical arrows*). Capillaries originating from the D‑AT have penetrated the cord substance (*transverse arrow*)

In order to illustrate that initial spinal cord blood supply in vertebrates is exclusively segmental, we present an anteroposterior (a.p.) view of a block of vertebromedullary segments together with the dorsal aorta from a 6-day-old chick embryo (Fig. 2a). Fig. 2b shows the beginning of intrinsic vascularization occurring from paired primitive anterior capillary tracts which are part of a perineural vascular plexus.

### 5–6-Week Cattle Embryo (Fig. 3)

**Fig. 3:**
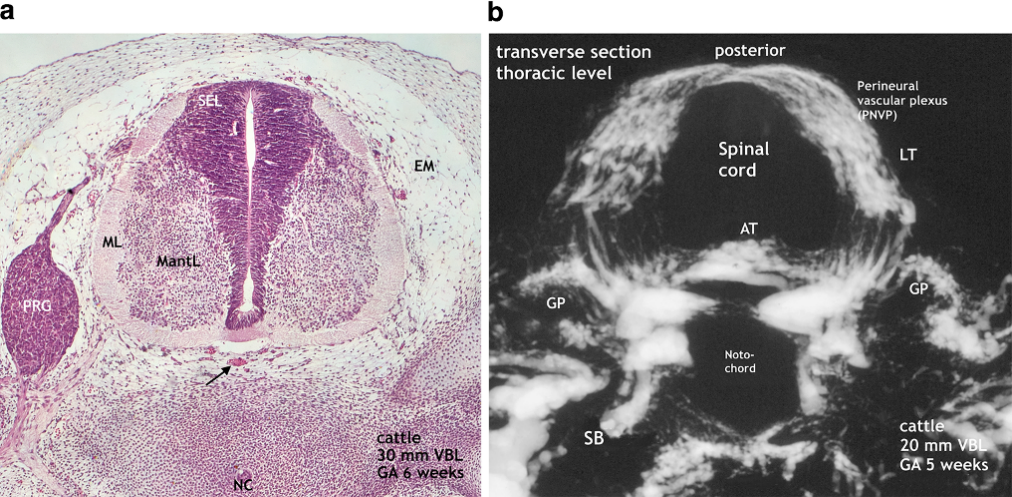
Maximum matrix formation. Perineural vascular plexus. Start of intrinsic blood supply of the embryonic cord. **a** Cattle, VBL 30 mm, 6 weeks GA. Histological section from a specimen at 6 weeks. Magnification × 100. The subependymal cell layer represents the germinal matrix and has its maximum extension in the spinal cord at that time. Contrary to the 5‑week specimen in **b** intrinsic capillaries intrinsic capillaries are clearly apparent. They penetrate the cord parenchyma from all parts of the surface, pass the marginal layer (ML) and mantle layer (MantL), and surround the border of the subependymal layer (SEL). Distinct blood vessels can also be observed within the primitive meninges (EM), in the anterior midline (*arrow*), and in other position. *PRG* posterior root ganglion, *NC* notochord. **b** Cattle, VBL 20 mm, GA 5 weeks. Microangiogram of a transverse section at thoracic level of a slightly younger specimen. This view reveals the structure of the network of capillaries enclosing the spinal cord. Circular components of the plexus are connected by anastomoses to form denser longitudinal formations, like the lateral tract (LT) or the paired or single anterior tract (AT). Further prominent structures include the bilateral ganglionic plexuses (GP). Supply is provided from the spinal branch (SB) of the segmental artery. The anterior tract (AT) is strong, large and uneven, insinuating a faint bilateral symmetry. So far, no intrinsic capillaries exist in this part of the cord

Our earliest observations concerning histology and microangiography in cattle were made in specimens 6 and 5 weeks old. On the microscopic section the subependymal layer or matrix has reached its maximum extension. Germinal cells originating from this layer will proliferate to form the basal and alar laminae resulting in the motoneurons (anterior horns) and the receptor cells of the posterior horns. This germinal matrix-phase lasts until the end of the second month. Internal vascularization within the neural tissue is evident in the microscopic image at 6 weeks (Fig. 3a). Capillaries are apparent in the marginal and mantle layers and reach the borders of the broad subependymal layer. Differentiation of the primitive meningeal membrane of the cord has not yet taken place. Similar to the chick embryo, a perineural vascular plexus (PNVP) covers the medulla of the cattle specimen but the microangiogram does not yet exhibit intrinsic blood vessels at 5 weeks (Fig. 3b). The circular parts of the PNVP are longitudinally interconnected by anastomoses. The resulting longitudinal tracts differ in density and extension on the medullary surface. This is shown for the broad lateral capillary tracts as well as for the anterior capillary tracts (AT) which may appear as a single, somewhat uneven formation as in Fig. 3a or a double tract (D-AT) as in the chicken (Fig. 2). On sections of the lumbar enlargement, the unpaired anterior tract (AT) is further developed and could already be called the primitive lumbar anterior spinal artery (not shown). The posterior root ganglia are always early and well delineated by an intense ganglionic plexus (GP).

### 7–8-Week Embryo (Figs. 4–7)

**Fig. 4:**
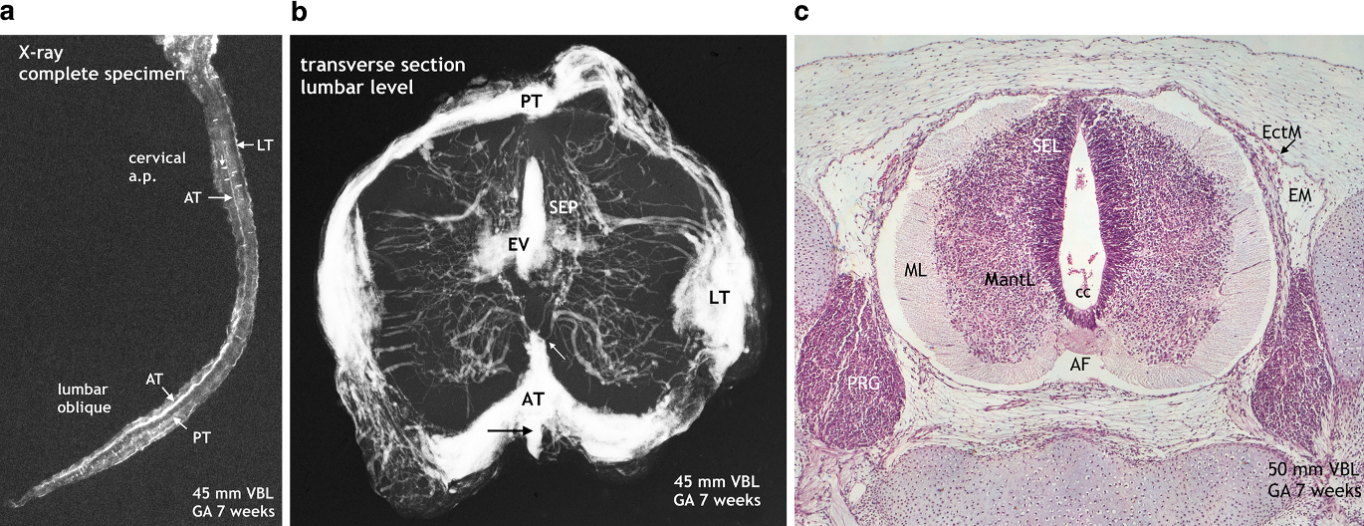
Intrinsic vascularization in the late embryonic stage. Development of the meninx secundaria. Cattle, VBL 45 mm, GA 7 weeks. **a** X-ray of the full injected spinal cord. In the lumbar region superficial formations (AT, LT, PT) look like compact tracts with blurred margins. This is in contrast to defined blood vessels in the cervical region but this aspect is caused by a considerably lower volume and extension of similar tracts at cervical level (not shown). Additionally, scattered transversely aligned islands of capillary tissue (*small vertical arrow*) on the surface of the enlargements. **b** Microangiogram of a transverse section at lumbar level. Broad and thick layers of superficial vascular plexus in the form of longitudinal tracts cover most parts of the cord surface, less compact on the posterior surface (PT) than in anterior and lateral position (AT; LT). The circumscribed ridge in the anterior midline (*arrow*) represents on additional sections an underlying anteromedian tract covered by the thick vascular plexus at the entrance to the anterior fissure (not illustrated). The majority of intrinsic vessels is derived from the lateral tracts. Most but not all of these contribute to the subependymal plexus (SEP). The penetrating vessels from the anterior tract (AT) mostly originate from the tip of the anterior fissure and are distinctly separate for the right or the left hemicord (*small arrow*). Others from the anterior and anterolateral surface take an immediate centrifugal course to the developing anterior horn area. Extravasation (EV) of contrast medium in the central canal and SEP. **c** Histology of a corresponding cattle specimen. VBL 50 mm, GA 7 weeks. Magnification × 80. The subependymal layer (SEL) or germinal matrix is already declining. Blood vessels in connection with the lateral surface pass the marginal layer (ML) to reach the mantle layer (MantL) or the circular subependymal vascular plexus. The primitive membranes of the cord have now been developed into an ectomeninx (EctM) or dura mater (*arrow*) and an inner vascular layer of endomeninx (EM) or meninx secundaria

**Fig. 5:**
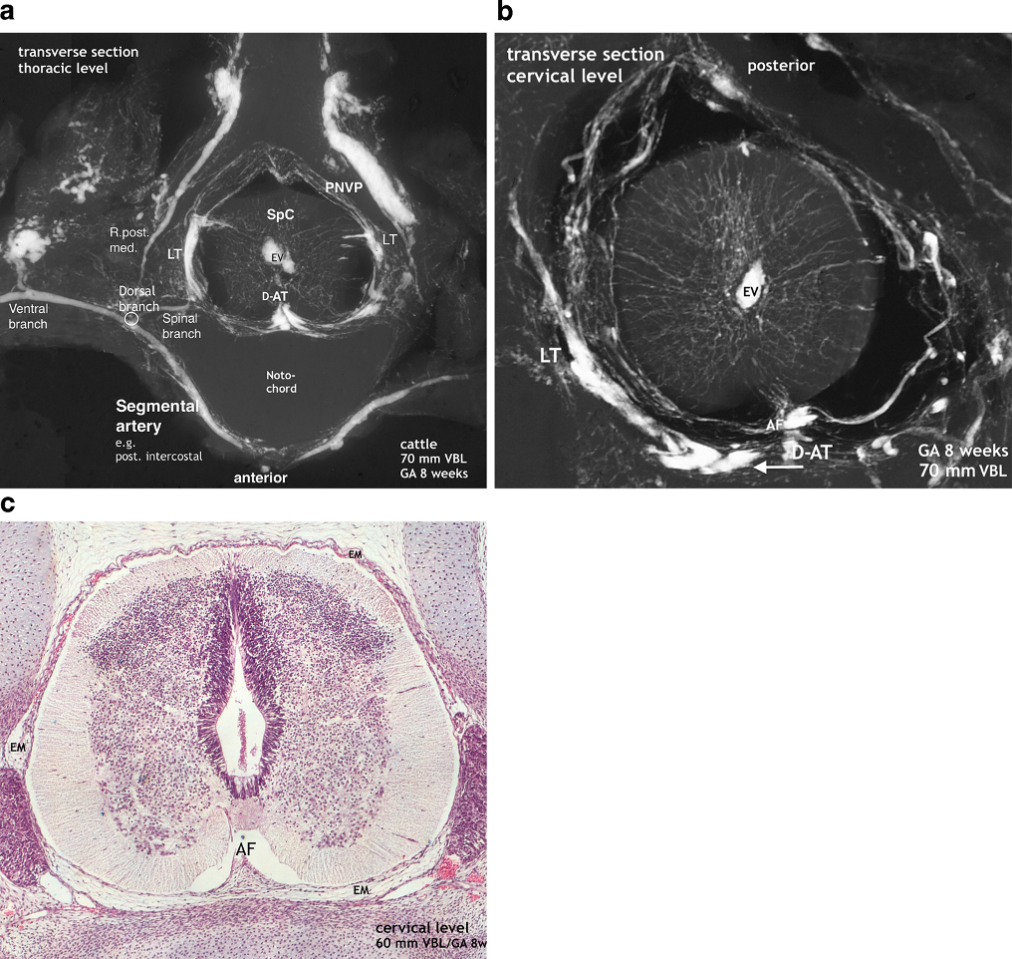
Decreasing germinal matrix phase. Vertebral column including spinal cord and secondary meninges. Cattle, VBL 70 mm, GA 8 weeks. **a** Microradiograph of a thoracic transverse section of an injected specimen. Segmental supply is provided by the spinal branches (SpB), assigned for the blood supply of the vertebral canal and its content, namely roots, meningeal membranes and the spinal cord. The secondary meninges contain thin circular layers of capillaries on the posterior surface as well as compact formations in the lateral tracts (LT). Capillaries from all parts of the surface, predominantly from the LT, have penetrated the medullary tissue centripetally to join a rather weak subependymal plexus in a thoracic matrix zone. The bi-parted or doubled flat anterior tract (D-AT) appears to be the precursor stage of primitive arterial channels. Penetrating capillaries arise from both tracts and course centrally on both sides of a virtual anterior midline in the corresponding hemicord. **b** Cervical level of the same specimen as in Fig. 7a. Typical embryonic pattern of the matrix phase. Equally sized intrinsic blood vessels join the central vascular plexus, established parallel to the decreasing subependymal layer (matrix). In the cervical region the vascular density of the plexus is higher compared to the thoracic cord. The doubled anterior tract (D-AT) shown at thoracic level in **a** has been displaced artificially during dissection at cervical level (*arrow*), leaving behind a smaller residuum of capillary tissue in the still shallow anterior fissure (AF). It gives rise to small parenchymal perforators directed posteriorly. (*EV* Extravasation). **c** Microscopic image at cervical level. Cattle, VBL 60 mm, GA 8 weeks. Magnification × 100. Progress in histogenesis with beginning formation of the posterior horns. The posterior part of the central canal has become narrower, the volume of the ependymal layer is diminishing, the marginal layer increasing in size. Note the gradual deepening of the anterior fissure (AF) and the extensive vascularization of the meninx secundaria or endomeninx (EM) covering the posterior and lateral surface

The cattle specimens of this series presenting radiological evidence of intrinsic medullary vascularization have already reached a gestational age of 7 weeks (Fig. 4). Initial intrinsic vascularization is very much adjusted to the demands of cellular proliferation and differentiation at the border zone to the subependymal layer (matrix) leading to the formation of a circular subependymal plexus. Histological sections comparing 6‑week-old and 7‑week-old embryos show not only progress in intrinsic vascularization but also in the development of the meninges. At 7 weeks, the initial primitive membrane of the cord has differentiated into an outer layer of dura mater and an inner layer of meninx secundaria containing the superficial blood vessels (Figs. 3 and 4). Mechanical displacement of vascular tracts is possible as shown in Fig. 5b. A strong double anterior capillary tract at the thoracic level (Fig. 5a) has become displaced at cervical level, leaving behind a residuum of capillary plexus in the flat anterior sulcus (Fig. 5b). The majority of early intrinsic capillaries at lumbar level originate from broad and thick lateral tracts, followed by the intrafissural compartment of the anterior tract. The posterior tract is comparably flat. As indicated in Fig. 4a and 5b the development of the cervical region lags behind, but the distribution of superficial vascularization is similar. Most, but not all, of the primitive vessels are directed centripetally toward the circular subependymal plexus. Others ramify in the matrix zone of the developing anterior and posterior horns. With respect to the principle of bilateral symmetry, it should be noted that the capillaries arising from the anterior tract near the apex of the anterior fissure appear to be sharply separated by a virtual midline and thus are confined to one or the other hemicord.

**Fig. 6:**
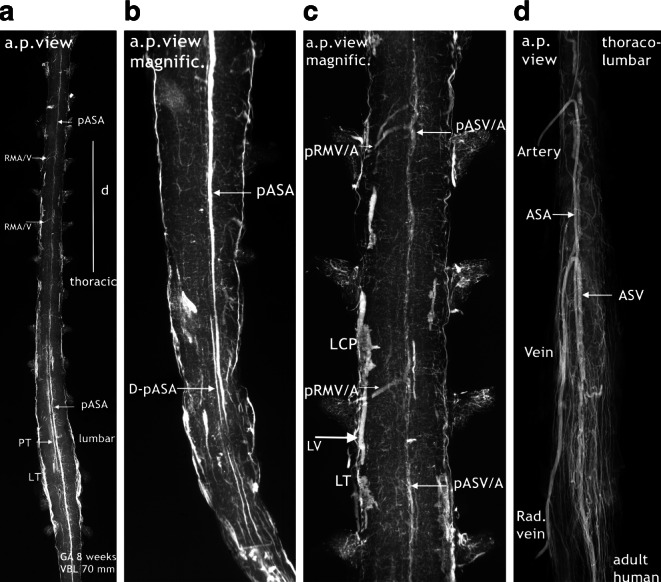
Special features in the superficial vascularization of a spinal cord at the end of the embryonic period. Same specimen as in Fig. 7. Cattle, VBL 70 mm, GA 8 weeks. Similar course and configuration of the anteromedian artery or vein in the adult. **a** X-ray survey of the lower two thirds of the injected spinal cord in a.p. view. The well-developed primitive anterior spinal artery (pASA) of the lumbar enlargement as well as lateral tracts (LT) become weaker at low thoracic levels. The line projected parallel to the pASA is a posterior tract (PT) and not part of a double pASA. At thoracic levels the pASA cannot be followed as a continuous blood vessel of regular contour and caliber. Nevertheless, several blood vessels looking like radiculomedullary arteries or veins (RMA/V) are visible at mid-thoracic levels. **b** Magnification of the cone reveals a persistent double primitive anterior spinal artery (D-pASA) at the coccygeal end of the spinal cord. **c** The position of this magnified thoracic section is marked by a *vertical line (d)* in **a**. At two segmental levels of the mid-thoracic region, a primitive radiculomedullary vein or artery (pRMV/A) of considerable size has been established, connected to an equally large-sized part of a primitive anterior spinal vein or artery (pASV/A) located in the midline. Identification of these vessels as arteries or veins is uncertain, but a strong arterial feeder in this location is unusual. Furthermore, a distinct lateral vein (LV) of large size (*thick arrow*) can be seen between patchy areas of local capillary plexus (LCP). **d** On the ventral surface of an injected adult human specimen, arterial and venous radiculomedullary blood vessels are similarly configurated blood vessels and the anterior spinal artery (ASA) and vein (ASV) are running close together above the entrance of the anterior fissure

**Fig. 7:**
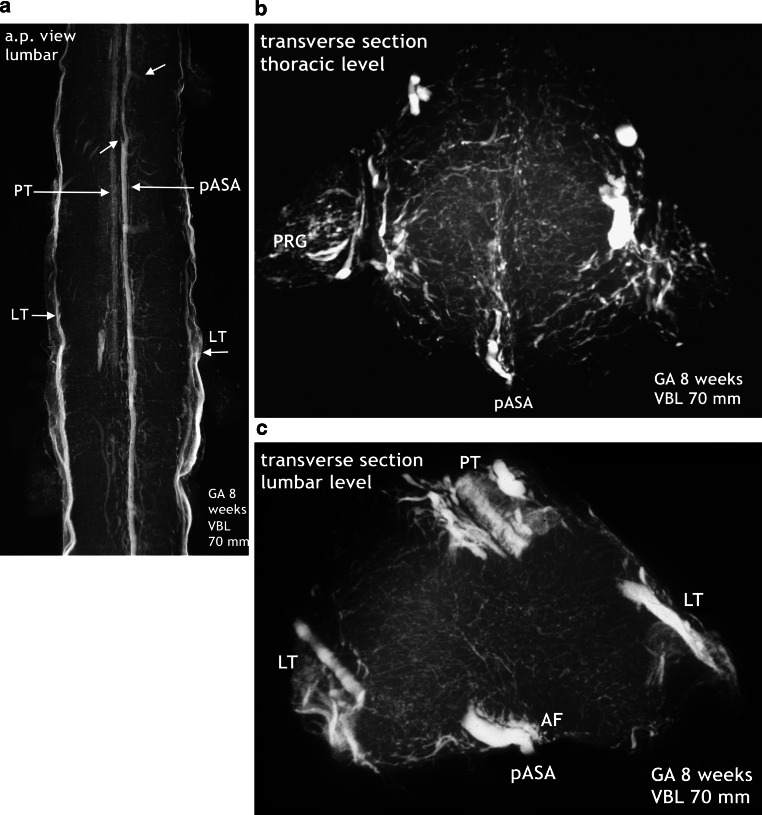
End of the germinal matrix phase. External and intrinsic spinal cord vascularization in different regions. Cattle, VBL 70 mm, GA 8 weeks. Same specimen as in Fig. 6. **a** X-ray of an injected spinal cord in a.p. view. Single primitive anterior spinal artery (pASA) of considerable size. Vascular sprouts at sites of changing caliber, but without connection to feeders (*oblique arrows*). Two almost symmetrical lateral tracts (LT) composed of capillary plexus and distinct, probably venous blood vessels. A less defined median posterior tract (PT) is projected parallel to the pASA. **b** Microangiography of a section at thoracic level in oblique axial view. Comparably weak and irregular pASA in paramedian location. Equally distributed intrinsic capillaries, typical for the late embryonic pattern. The superficial network is composed of rather loose meshes with occasional capillary islands. A cervical microangiogram of this specimen is the one shown as Fig. 12a. It differs in the larger amount of superficial capillary tissue and the configuration of the thin anterior arterial tract. The poor presentation of the craniad of the lumbar intumescence may be a sign of their beginning regression (compare Fig. 9). **c** Microangiogram of a transverse section in an oblique axial view. The primitive anterior spinal artery tract (pASA) is accompanied by triangular formations of capillary plexus extending into the anterior fissure (AF). Provisional small sulcal arteries have developed from the infolded meningeal formations. The strong longitudinal tracts in lateral and posterior position contain not only laminar and focal capillary plexus, but also distinct vascular channels. The intrinsic vascularization pattern is typical for the embryonic phase

The end of the embryonic period coincides with the termination of the matrix phase. The matrix layer is widely used up and the subependymal intrinsic plexus has disappeared. Vascularization of the nervous tissue in the thoracic and lumbar regions is now homogeneous, but not beyond the normal range (Fig. 7b, c). On the surface of the cord formation of a primitive anterior spinal artery (pASA) is evident, well-developed in the lumbar region (Fig. 6a); however, in the thoracic and cervical regions first signs of regression have to be noted (Fig. 6a and 7b). These circumstances may cause problems in the evaluation of some well-injected specimens as in the 8‑week specimen demonstrated in Figs. 6 and 7. The findings at the lower end are unambiguous since transverse sections can be involved in the evaluation. They confirm a strong lumbar pASA continuing as a double artery in its further course to the sacrococcygeal end of the medulla, a finding we could observe repeatedly. A clear assignment is also possible for an unusually strong posterior tract and lateral tracts including venous blood vessels; however, the classification of the impressive radiculomedullary blood vessels in the thoracic region is unclear (Fig. 6c). The differential diagnosis between the preliminary version of a radiculomedullary artery or its venous counterpart cannot be decided (Fig. 6d). Arguments against a primitive arterial feeder are that the pASA is already in regression at this level and that arteries in this section of the cord are generally small in size. In the absence of an angiographic series which provides a separation of arterial and venous phases we may not be able to differentiate these vessels on the anterior surface on the basis of size, location or configuration (compare Fig. 6d).

### 9-Week Fetus (Figs. 11c, 12f, 13a)

**Fig. 8:**
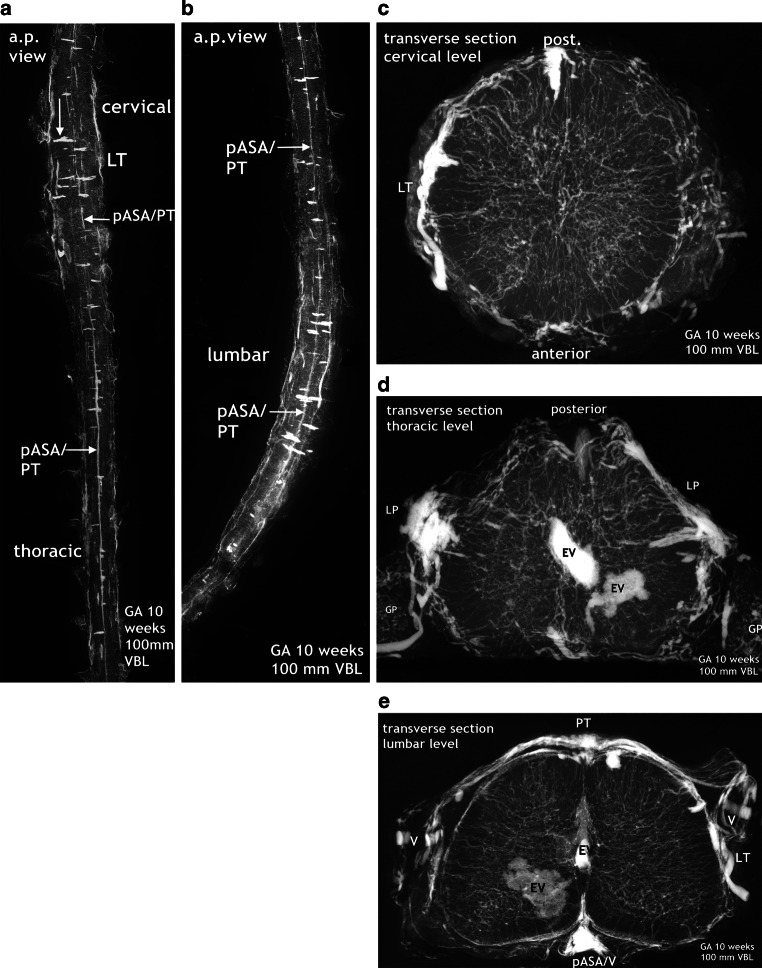
Beginning reorganization of the embryonic vascularization pattern. Regression of the primitive ASA, appearance of intrinsic hypervascularity and superficial vascular tissue including the numerous horizontally aligned capillary islands. Cattle, GA 10 weeks, 100 mm VBL. Same specimen in all parts of the figure. X‑rays of an injected complete spinal cord. **a** The pASA has disappeared from the cervical spinal cord. What looks like residual sections are rather parts of capillary tissue in posterior tracts (PT), as seen on the transverse section in **c**. Discontinuous lateral tracts (LT) and transverse stripes of capillary islands (*vertical arrow*) predominate. Thoracically, the linear midline structure may represent a thin anterior artery or a posterior tract, when compared with the transverse section illustrated in **c**. **b** The vascular status of the lumbar enlargement is similar to the cervical one (**a**). Preserved parts of the previous lumbar pASA look ill-defined and irregular. **c**,**d** Not well-defined or only small pASA. The density of equally sized intrinsic capillaries is high. All vessels are penetrating from discontinuous layers of capillary plexus, which cover mainly the lateral surfaces as fragmented tracts (LT) or local plexus (LP). *GP* ganglionic plexus, *EV* extravasation. **e** Complex presentation of a residual lumbar section of the pASA. Possibly additional overlap with surrounding capillary tissue. Typical embryonic pattern of intrinsic vascularization with a more pronounced difference in vascular density between grey and white matter. On the lateral and posterior surfaces, distinct blood vessels of the venous type have developed together with layers of capillary plexus

**Fig. 9:**
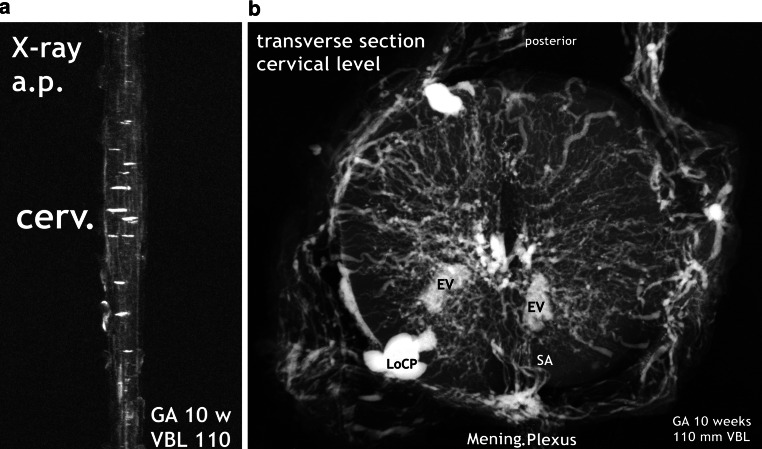
Burst of vascularization. Phase I. Massive increase of circumferential vasculature and perforating branches of the peripheral system. **a** X-ray film of the injected cervical spinal cord in a.p. view. The most striking features are the many islands of capillary plexus aligned transversely on the surface of the cord and the obvious regression of the primitive anterior spinal artery. **b** Microangiogram from the cervical region. The thus far regular and uniform size and distribution of superficial and deep arteries has now become an aspect of relatively heterogeneous and immature arteries and massive hypervascularization. Scattered formations of local capillary plexus (LoCP) on the surface add to the somewhat chaotic image. The primitive ASA is replaced by an irregular vascular plexus from which moderately sized sulcal arteries (SA) arise

The observations in 9‑week fetuses may be similar to the beginning reorganization as demonstrated in Fig. 8. In this case regression of the pASA goes along with the appearance of irregular formations of capillary plexus in addition to horizontally aligned capillary islands on the enlargements. Changes may also be more advanced with a beginning burst of vascularization and prominent intrafissural sulcal arteries emerging in the deep anterior median fissure (Figs. 9, 11c, 12f). Histologically, regression of the subependymal layer around the narrowed central lumen confirms the end of the matrix phase at 9 weeks (Fig. 13a).

### 10-Week Fetus (Figs. 8–14)

The signs of forthcoming changes in the vascular architecture can best be studied on the X‑rays and microangiograms shown in Fig. 8. Discontinuation and fading of the longitudinal channels are accompanied by the appearance of many transversely aligned capillary islands scattered across the surface of the intumescences (Figs. 8 and 9). Lateral and posterior tracts of capillary tissue also increase in size and extension. In the lumbar cord, discontinuous lateral tracts include defined vascular channels, most probably veins of different size (Fig. 8e). The moderate density of intrinsic vascularization, the small vessel size, and the uniformity of vessel distribution still correspond to the embryonic pattern. In the next step of development, exemplified by the specimen shown in Fig. 9, intrinsic vascular density has significantly increased. The elongated blood vessels vary in size but keep the common centripetal course of peripheral perforators. No anterior spinal artery can be defined on the X‑ray survey. Instead, a plexiform formation is giving rise to somewhat enlarged and elongated sulcal arteries.

**Fig. 10:**
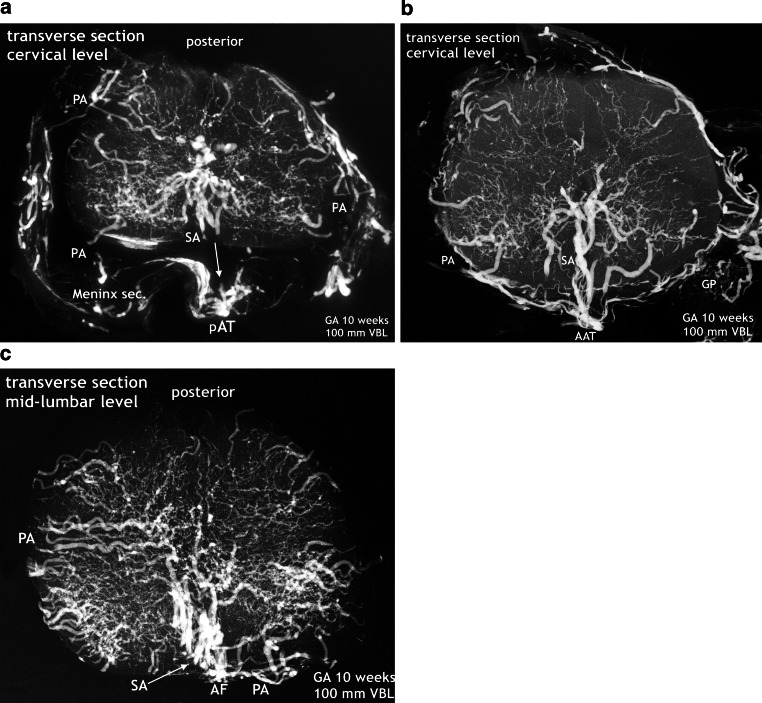
Burst of vascularization. Phase II. Explosive emergence of large blood vessels inclusive of sulcal/central arteries. Transverse sections in different regions of three injected specimens. Cattle. GA 10 weeks, 100 mm VBL. Transverse sections from the cervical, thoracic lumbar region of three different specimens. The primitive meninges could not always be preserved. **a** Cervical level. Derived from the meninges of the entire surface area, an enormous number of large blood vessels have penetrated the nervous tissue of the cord. Displacement of the secondary meninx mainly from the previously infolded entrance of the anterior fissure (*vertical arrow*). Several sulcal and peripheral arteries (PA) have been torn off from the meningeal layer. Sulcal arteries of this large size had not previously existed. The vessels turn to one side, enter the parenchyma and ramify as central arteries (CA) in the anterior horn area. Regression of the pAT to an insignificant vascular channel is clearly apparent. **b** On this transverse section at cervico-thoracic level superficial capillary plexus is replaced by distinct peripheral blood vessels (PA). Even larger branches of these enter the medulla directly from the convexity. Others follow the intrafissural space as sulcal arteries (SA). An ASA or anterior anastomotic tract (AAT) cannot be reliably defined in this plane. **c** In this early phase of explosive vascularization of the enlargements, the multiplicity of unbranched large blood vessels appears completely disproportionate. Contrary to the advanced distribution pattern in **a** and **b**, the basic design of a future system of central and peripheral supply is not yet apparent

**Fig. 11:**
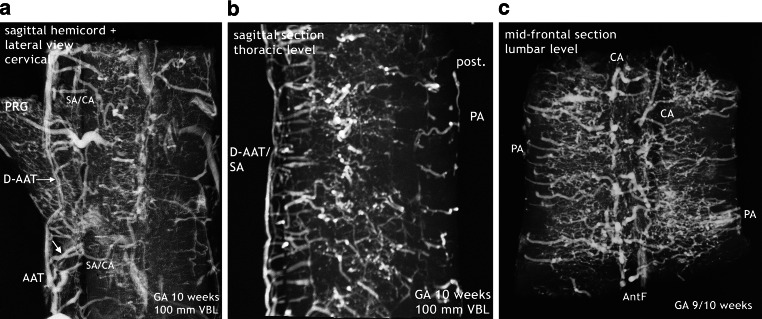
Burst of vascularization. Phase II. Return to the “anlage” of bilateral symmetry demonstrated on sagittal and frontal sections. Cattle, GA 10 weeks, 100 mm VBL. **a** Cervical level of the same specimen as in Fig. 14a. The sulcal/central arteries arise in two lines from a doubled anterior anastomotic tract (D-AAT) or from a single tract (AAT) with a short common trunk (*oblique arrow*). Posterior root ganglion (PRG). **b** Mid-sagittal section of the thoracic region. Same specimen as in Fig. 10b. Two thin anterior channels of somewhat different size connect the two dense rows of sulcal arteries longitudinally (D-AAT/SA). The middle part of the image represents the ramification area of central and peripheral arteries. Peripheral arteries penetrating from the posterior surface (PA) are less numerous. **c** Different specimen. Mid-frontal section through the anterior horn area at lumbar level. Central and peripheral arteries meet and branch preferentially in this zone, forming a dense network. Side-alternating course of central arteries (CA) in the anterior fissure (AntF). The disordered distribution of central (CA) and peripheral artery branches (PA) reflects the burst of vascularization (compare Fig. 1d)

**Fig. 12:**
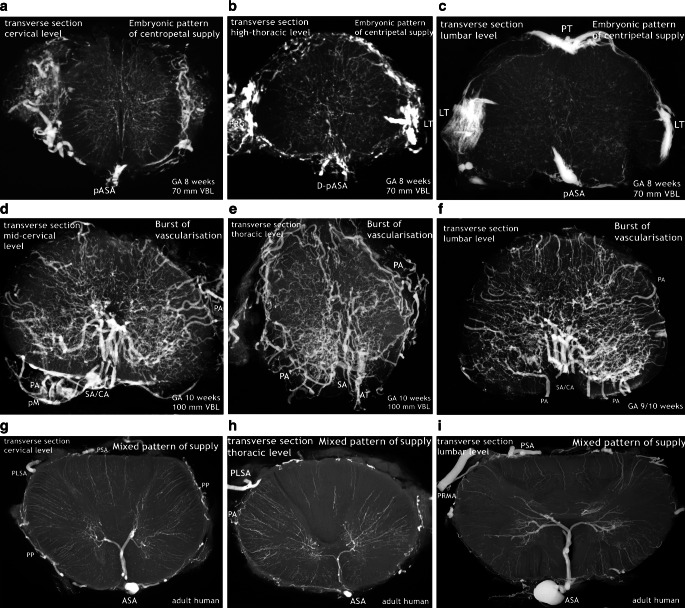
Changing pattern of medullary blood supply. Embryonic phase (*top row*), burst of vascularization in the early fetal period (*middle row*). The bottom row shows the final architecture in an adult human specimen for comparison. Transverse sections of the cervical, thoracic and lumbar regions. Cattle, GA 8–10 weeks, 70–100 mm VBL. **a**–**c** The embryonic pattern at the end of the germinal matrix phase is characterized by a double (D-) or single primitive anterior spinal artery (pASA) as shown in the cervical and thoracic cord and equally distributed and centrifugally oriented intrinsic capillaries. The pASA at lumbar level develops as a strong unpaired vessel at an early stage. On the lateral surfaces longitudinal tracts (LT) may be composed of capillary plexus or of mixed formations of vascular tissue, including definite vascular channels. A strong posterior tract (PT) is only apparent on the lumbar enlargement. **d**–**f** The burst of vascularization creates the pattern for additional centrifugal supply. An overshoot of newly formed blood vessels from the meningeal surface supersedes the embryonic pattern. Vessels appear in complex, occasionally bizarre configurations, at lumbar level accentuated in the ventral two-thirds of the cord. Sulcal arteries (SA) to the hemi-cords arise in abundance from the entrance of the anterior fissure. **g**–**i** The adult pattern: A central (centrifugal) system and a peripheral (centripetal) system share in the supply of the cord substance. The ASA is origin of branches to the central system (SA/CA) and of branches for peripheral supply in the ventral two thirds of the cross-sectional area. Posterior feeders (PRMA) and posterolateral tracts (PLSA) supply the posterior horns and the posterior third of white matter (**g**–**i** Reproduced from [17])

The major increase in vascularization culminates in a burst of vascularization affecting the entire spinal cord (Fig. 10). Although displacement of the secondary meninges often happens by cutting the spinal cord tissue, parentage of the blood vessels from the secondary meninges is obvious on undamaged sections. Large branches of the peripheral system penetrate the nervous tissue and only the posterior area is relatively spared. The blood vessels are directed towards the gray matter, especially the area of the anterior horns. At the same time, two rows of large-sized vessels have appeared between the meningeal layers of the anterior fissure. These true sulcal arteries lie close together. In the depth of the anterior fissure they turn to one side or the other, penetrate the cord tissue and then follow a centrifugal course (Figs. 10 and 12). The burst of vascularization produces an enormous transient overshoot of peripheral and sulcal/central arteries. Occasionally, chaotic or bizarre blood vessel configurations and distributions are observed (Fig. 12d,e). In this phase of vascular overshoot the determination of the exact origin of the new sulcal arteries may be uncertain on transverse sections but, as shown on sagittal sections, the sulcal arteries are anastomosing on each side among themselves, thus forming small-sized longitudinal anastomotic tracts at the entrance of the anterior fissure (Fig. 11a,b and 14b). This means that the developmental process has transiently returned to the primary disposition of bilateral symmetry. In one specimen, this beginning reconstitution of an anterior spinal artery is evident at upper thoracic level. The microangiogram shows a hemicord including the anterior midline area in a.p. view (Fig. 14a). The premature blood vessel is partially duplicated and exhibits narrow segments and extensive irregularities of caliber. This specimen additionally suggests superficial tracts in the position of the later posterolateral and posterior spinal arteries (PLSA, PSA). The contrasting appearance of the embryonic vascularization pattern at about 8 and 9 weeks, the burst of vascularization around the 10th week and an adult pattern of supply illustrate the fundamental reorganization of the vascular supply pattern in Fig. 12.

**Fig. 13:**
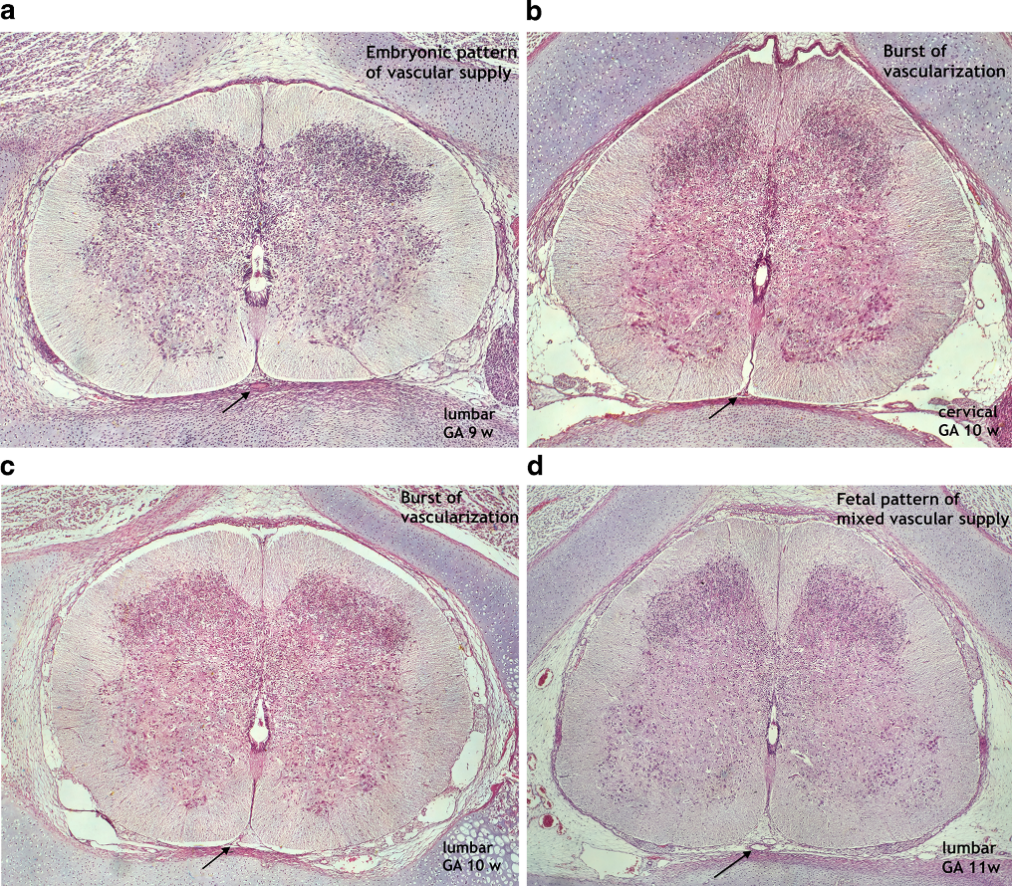
Findings in histological sections before, during and after restructuring of the medullary arterial supply. Cattle specimens 9, 10, 11 weeks GA; 83, 99 and 121 mm VBL. Magnification × 80 (a, b), × 60 (c, d). To illustrate the explosive increase in vascularization at different levels, sections from the cervical and lumbar region have been selected from the 10-week specimen. Hematoxylin-eosin (HE) staining. **a** The matrix phase is close to completion, the residual cell layer in the subependymal zone has become small. Progressive formation of the anterior and posterior grey columns. Well-developed midline blood vessels at the entrance to the anterior fissure correspond to a single primitive anterior tract (pAT) and to vascular tissue in the endomeninx. Unremarkable distribution of small intrinsic blood vessels. **b**,**c** Substantial increase in motor cells in the developing anterior horns and of receptor cells in the posterior horns. Increasing gray matter-white matter ratio and changing configuration of the grey columns with deepening of the anterior fissure. Major increase in internal and external vascularization. Symmetrically paired vascular cross sections at the entrance to the anterior fissure at cervical level (**b**, *arrow*), excentric single vessel paramedian of the anterior midline at lumbar level (**c**, *arrow*). **d** Further development of the gray matter and white matter tracts. Shape and morphological pattern of the spinal cord approach the adult form. The pial process of the anterior fissure now demonstrates cross-sections of two large and several small blood vessels. The histological aspect of the former resembles that of an anterior spinal artery and an anteromedian venous trunk. Abundant superficial vasculature in the meninx secundaria and first appearance of large veins in the epidural space

The histological sections of these time intervals vividly document the progress of the developing spinal cord with the major increase in the motor cells of the developing anterior horn and, to a lesser degree, of receptor cells of the posterior horn (Fig. 13). In addition, at the entrance of the anterior fissure different cross-sections of blood vessels can be identified during these two periods: a primitive anterior tract in the embryonic period (a), very small paired tracts (b), and an ill-defined paramedian tract (c) in the phase of remodeling. In every case, an extensive vascular plexus is apparent within the meninx secundaria.

### 11-Week Fetus (Figs. 14–16)

Following the burst of vascularization major changes can be observed: regression of the vascular overshoot, the beginning implementation of additional central supply and the reappearance of a large arterial trunk on the anterior midline. Histologically, the morphological pattern of the spinal cord including the differentiation of meninges and vascularization approaches the adult form.

**Fig. 14:**
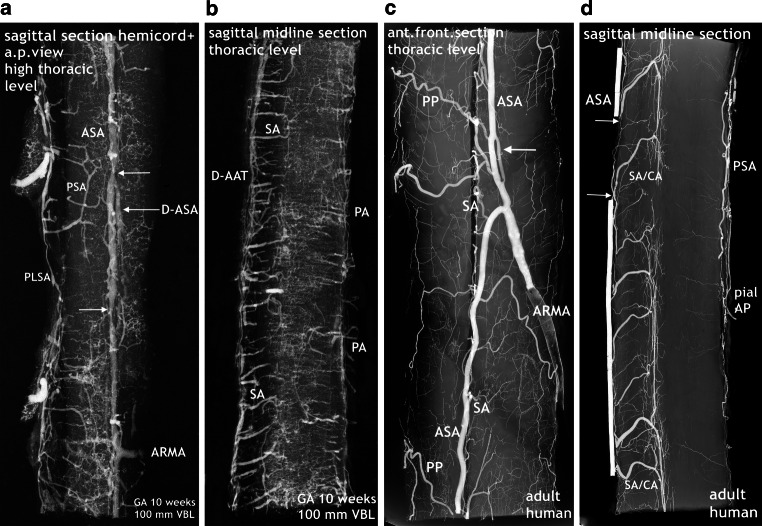
Restructuring of arterial supply with beginning re-establishment of an unpaired anterior spinal artery (**a**,**b**). Cattle, GA 10 weeks, 100 mm VBL. Same specimen as in Figs. 10a, 11a, 12d, e. Comparison with adult human supply patterns (**c**,**d**). **a** Microangiogram in a.p. view. Sagittal section of the right half of a cord at high-thoracic level including the redeveloping single anterior spinal artery (ASA). Blurred margins, narrow sections and residual duplications (D-ASA; *arrows*) characterize the still immature artery. Confluence of an anterior radiculomedullary artery (ARMA) is suggested in the lower third of the vessel course. In the lateral and posterior part of the hemicord blood channel configurations look similar to the later posterolateral and posterior spinal arteries (PLSA, PSA). **b** Microangiogram of a mid-sagittal section from a lower thoracic level of the same specimen as in **a**. At this level the double anterior anastomotic tract (D-AAT), having temporarily returned, is still present as anastomosis between the immature sulcal arteries (SA) on both sides. The SA take an almost horizontal or slightly ascending course. Peripheral arteries (PA) penetrate the posterior columns. **c** Microangiogram of the anterior frontal section of the spinal cord in an adult human. A more or less pronounced “hairpin” arrangement between the ascending radiculomedullary artery (ARMA) as a contributor and the descending ASA branch is frequently observed. The confluence may be located apart from the midline. Duplication persisted in a short segment of the ascending branch of the ASA in this case (*arrow*). The ASA gives not only origin to sulcal arteries (SA), but also to peripheral arteries of the ventral two thirds of the pial arterial plexus (PP). **d** Mid-sagittal section of thoracic level. The ASA acts as a longitudinal anastomosis along the entire length of the spinal cord. Note the low number of SA in this region and the extensive vertical branching of the CA near the central canal. At the entrance of the anterior fissure interconnections between several sulcal/central arteries below the level of the ASA can be observed (*arrows*). (**d** Reproduced from [17])

**Fig. 15:**
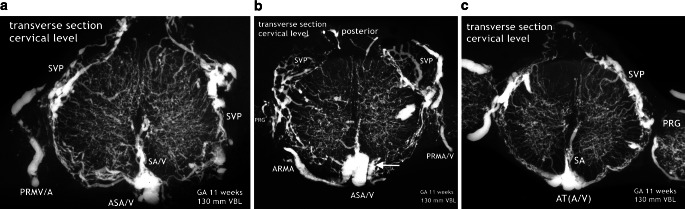
Implementation of a system of bidirectional supply, reformation of the anterior anastomotic tract, and segmental feeders. Cattle, GA 11 weeks, 130 mm VBL. All sections 1 mm thick. Possible co-imaging of concomitant veins is termed as “/V”. **a** A novel anteromedian arterial tract has appeared, presenting as a large vascular structure that covers the entrance of the anterior fissure. This tract, the anterior spinal artery (ASA), is the origin of large sulcal/central arteries (SA/CA). The increase of central and peripheral arteries results in a dense arrangement of intrinsic blood vessels. Supply from the periphery (centripetal) is still predominant. A systematic pattern of individualized posterior tracts has not yet developed from the superficial vascular plexus (SVP). Posterior radiculomedullary vein/artery (PRMV/A). **b** Large anterior radiculomedullary artery (ARMA) joining the ASA. Plexus-like vascular elements in the anterior midline close to the ASA (*arrow*). The superficial vascular plexus (SVP) in lateral and posterior position still appears as a disordered coarse mesh. **c** Extensive anterior tract. Long sulcal blood vessels (SA/V), indicating increasing central supply. But the vascular perfusion pattern of the whole cross-sectional area is still dominated by centripetal vessels from the surface. Vascular density differs very much between central gray and peripheral white matter. Prominent superficial vascular plexus (SVP) on the posterolateral surface. Posterior root ganglia (PRG)

**Fig. 16:**
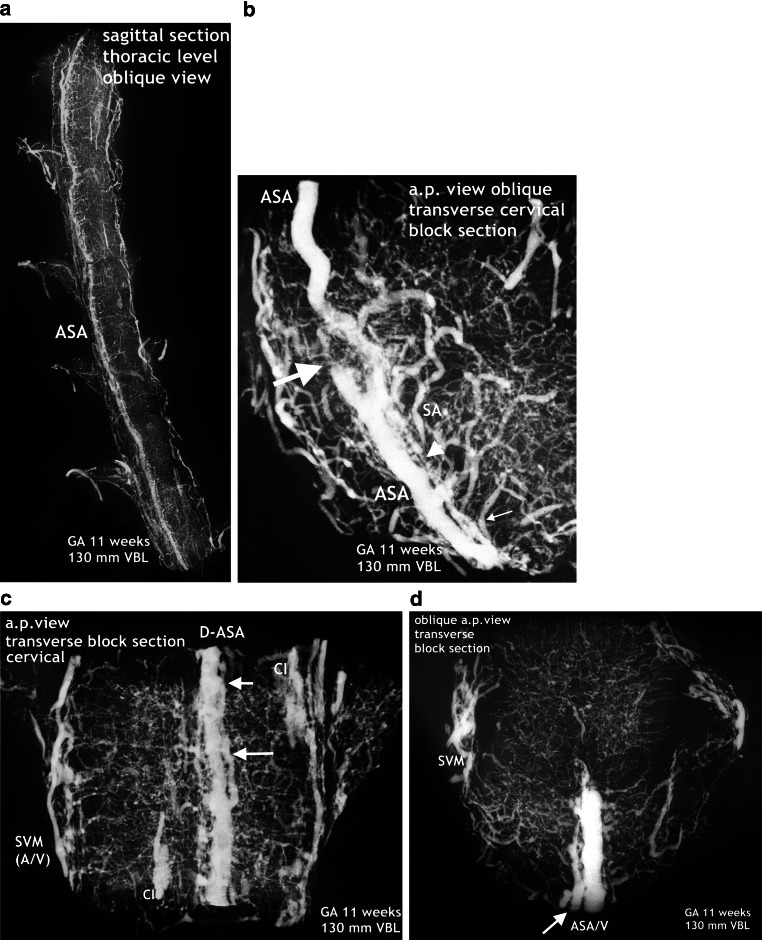
Development of the definite anterior spinal artery. Cattle, GA 11 weeks, 130 mm VBL. Same specimen as in Fig. 15. **a** Defined anterior trunk, representing a large anterior spinal artery (ASA). Formation of a common single pathway was obviously not yet successful in all sections of the vessel course. **b** Anterior trunks developed on different sides, one ending blindly (*large arrow*). The other, carrying the sulcal arteries of this side (*small arrow*) becomes blurred before the vessel size decreases significantly. Presumably, the main channels will be connected to each other but incomplete regression of one channel over a short distance might be an additional outcome. **c** Segmentally paired channels of unequal size (*long arrow*) with unification (*short arrow*). The lateral superficial vascular mesh (SVM) includes distinct longitudinal channels serving for supply or drainage (A/V). Additionally, scattered vertical capillary islands (CI). **d** The large ASA channel is accompanied by a smaller one corresponding to a possibly persisting not uniting small channel (*arrow*)

**Fig. 17:**
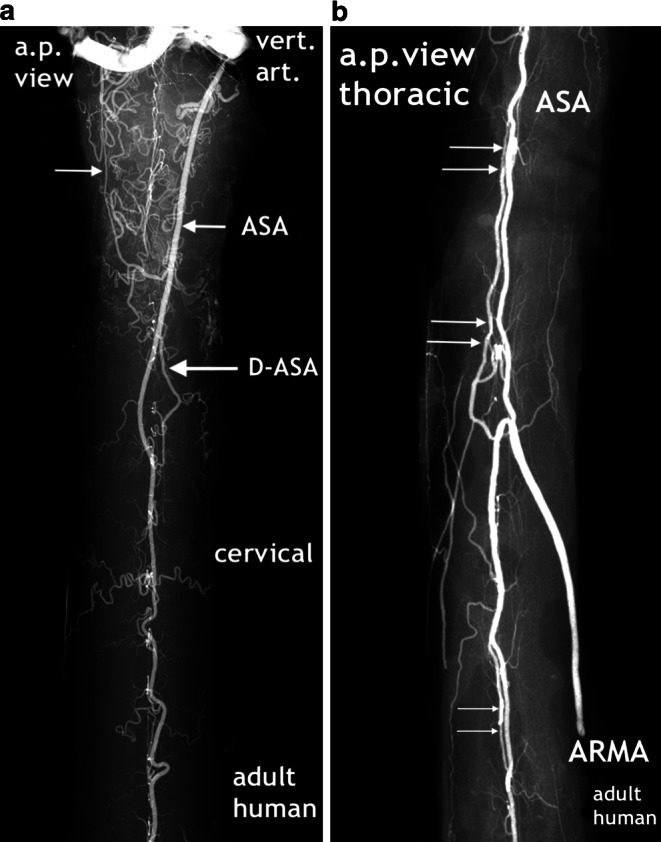
Embryologically defined variations of the anterior spinal artery in adult humans. **a** The symmetrically paired origin of the anterior spinal artery (ASA, *small arrow*) should be the rule, but instead it is rather exceptional. In this case the anterior midline trunk is formed solely by the descending branch of the left vertebral artery. When the vessel reaches the midline at the C1/2 level, it continues as two channels of unequal size, the stronger being paradoxically the contralateral right one. After a short course with lateral deviation of both tracts from the midline (*large arrow*, D‑ASA), the tracts converge again, but do not unite. What looks like formation of an island is pseudo-island, because the smaller artery terminates as a sulcal/central artery in the anterior fissure. On the right side, a hypoplastic descending branch terminates in a network of tortuous, superficial arteries covering and supplying this half of the medulla oblongata (*small thin arrow*) (Reproduced from [17]). **b** Duplication of the anterior spinal artery, better persistence of a paired “anlage” is based on the phylogenetic and ontogenetic disposition. The two sections of ASA-duplication in this example show in the more caudal manifestation a close parallel course of the channels (*small double arrows*). A stronger deviation of the tracts from the midline exists more cranially near the entry of a large feeder (*large double arrows*). The caliber of the two tracts is slightly different and unification may or may not occur (reproduced from [18])

The new, still immature but probably final version of an anterior tract is a wide channel that ultimately will form the anterior spinal artery, reinforced by selected radiculomedullary feeders (Fig. 16). Its definite course as a single trunk is once more based on two paramedian anastomotic channels which carry the sulcal/central branches to supply the corresponding hemicords (Fig. 14a,b and 15). The transient vascular overshoot has created a dense system of intrinsic blood vessels (Fig. 15a). The vascular density is now significantly different between the marginal layer and the area of the central gray matter (Fig. 15c). Branching of sulcal/central arteries (Fig. 15a) is developing but the peripheral system of perforating arteries originating from the secondary meninges is still predominant. As illustrated in Figs. 15 and 16 the re-establishment of an unpaired arterial midline-trunk may follow a side-alternating mechanism in which one of the two channels will be selected to become the leading anastomotic trunk. Alternatively, the paired primary channels can be included in a vascular midline formation of large size. Failures of forming a common trunk are demonstrated in Figs. 16 and 17. Anterior and posterior radiculomedullary feeders can be identified (Fig. 15), but development of a structured pial network on the posterior surface is still missing.

On the histological section of the 11-week fetus, differentiation of the anterior and posterior columns of gray matter and development of the white matter tracts are already similar to the adult configuration. Motor and sensory neurons are numerous. The appearance of intrinsic capillaries is more evenly distributed than before. They are less numerous in the white matter and more highly concentrated within the gray matter of the anterior horns. At 11 weeks (Fig. 13d) they have accentuated perivascular spaces, which facilitate the recognition of the different vessel courses. A large anterior spinal artery with a well-defined vessel wall is detectable in midline position within the meninx secundaria at the entrance to the ventral sulcus. The anterior median fissure has further deepened and contains numerous cross-sections of blood vessels, and a distinct larger vessel below the artery could be the anterior spinal vein.

### Adult Human Variations (Fig. 17)

These final images may only exemplify the clinical impact of these developmental variations that can be documented by X‑ray films of injected adult human specimens. Variations at the cranial origin of the ASA with asymmetric patterns of the descending branches are frequent findings as well as variable types of persistent paired anterior spinal arteries, mostly observed in the cervical and thoracic areas of the spinal cord.

## Discussion

This series comprises only a small number of cases within a limited interval of gestational age. On the other hand, the majority of our specimens underwent an examination of the entire spinal cord. In order not to neglect the regional characteristics of spinal cords we considered the common presentation of findings at cervical, thoracic and lumbar levels as an important contribution to an improved understanding of the development of the arterial supply to the spinal cord.

We cannot comment conclusively on the early embryonic development of the vascular pattern in the walls of the neural tube of mammals because we have no examinations of the spinal cord in cattle prior to 5 weeks; however, our experience here, although limited to 7 observations in cattle embryos and 3 in chicks, is in agreement with the detailed and thorough studies carried out at the end of the nineteenth and the beginning of the twentieth centuries of the earliest stages of internal vascularization and the establishment of a primary anterior tract [6, 8–10, 20, 22]. It should be stressed that during this period in scientific anatomical research the early embryonic period and comparative studies were the focus of much more interest than fetal development.

It is generally accepted that during the first weeks of the embryonic period, external supply to the spine and spinal cord is strictly segmental and in humans is accomplished by the dorsal branches of maximally 31 pairs of segmental arteries of the aorta ([23–25]; Fig. 2). This principle of bilateral supply of all components of a metamere from every level remains valid both prenatally and postnatally with the sole exception of the spinal medulla. The high degree of similarity in the vascular anatomy of this region among vertebrates is obvious when comparing the Fig. 2 with 3 and 5a.

Nutrition of the neural tube in the first weeks of embryonic life is based on diffusion from a perineural vascular plexus (PNVP, Fig. 3) lying within the meninx primitiva upon a single layer of epithelial cells [26, 27]. When histogenesis of the central nervous system (CNS) is beginning, the increasing metabolism of the embryo demands an adaptation of vascular supply [3, 28]. For the spinal cord this is achieved through the formation of compact tracts of superficial capillary tissue and the advent of intrinsic vascularization (Figs. 4 and 5). The capillary tracts develop predominantly in lateral position, moreover as a double or single tract anteriorly. The advent of intrinsic vascularization occurs in the chick embryo before 6 days of incubation from paired anterior capillary tracts, in higher mammals it progresses from a single formation not before the beginning of the second month ([29–31]; Fig. 3). The paired or single anterior channel is the developing primitive anterior spinal artery lying across the entrance of the flat anterior fissure.

The point of origin of the first capillaries penetrating the neural parenchyma was a matter of dispute for a long time [6, 8]. It culminated in the observation of a sudden appearance of total intrinsic vascularization in the rabbit [22] supporting the assumption of an additional intrinsic origin of capillaries independent from the superficial source [22, 32]. This hypothesis could not be substantiated by others [19, 20, 33, 34] and would have contradicted a fundamental law of angiogenesis formulated by Lierse [35] namely that vascularization of a tubular organ like the neural tube always occurs through radially penetrating shoots from a superficial capillary net. In their study of the earliest vascularization in the mouse, Nakao et al. [34] confirmed invading shoots from all three capillary tracts of the PNVP with a strong preponderance of the lateral tracts.

We observed an early origin from the double primitive anterior tracts in the chick (Fig. 2b) but in our 7‑week cattle specimens numerous blood vessels have already entered the cord substance from all parts of the surface. They course in a centripetal direction to a zone of subependymal cell layers that have developed around the central canal (Figs. 4 and 5). This proliferation zone or matrix is accompanied by a subependymal vascular plexus and a zone of cell differentiation [6, 20, 26, 36] Formation and regression of the matrix mark a period of tissue maturation that can be observed in all parts of the CNS, earliest in the spinal cord [3, 12, 35, 36]. At the end of the matrix phase, which coincides in spinal cords of cattle with the end of the 8th week, the subependymal plexus has mostly disappeared. Slowly but steadily progressing internal vascularization has resulted in an unstructured distribution of small vessels as the typical intrinsic embryological pattern. In the same period a more or less well-defined primitive anterior spinal artery has developed presenting on the lumbar enlargement as a strong single trunk (Fig. 7).

Development of the anterior median fissure is the result of the proliferative activity of the matrix and the subsequent cell differentiation due to the increasing volume of the anterior horn area. Kadyi [37] described the specific new role of the previously peripherally located meninges which have been passively converted into an inner surface. These simultaneous changes of gross morphology and metabolic demands in special areas of the cord are suitable for an adaptation of the vascular supply pattern. The enclosed double layer of the primitive meninges, the later processus anterior piae matris [37], not only offers an internalized path for originally peripheral blood vessels. It also contains all the properties of the meninges on the outer surface together with the “anlage” for paired arterial chains. This passive infolding of outer meningeal surface between the emerging tissue components of the developing spinal cord is morphologically different from the constitution of the choroid plexus of the brain; however, intraventricular displacement of meningeal surface in the brain as well has been described as an intermediate mode of supplementary nutrition to the neural tube by extrinsic supply, separating the prechoroidal and the choroidal stages [28].

The differences in X‑rays of injected specimens at 8‑weeks and 10-weeks gestational age (Figs. 7, 8 and 9) signalize that a significant change of the vascular supply pattern is on the way. Regression of the primitive anterior tract is almost complete and the many transversely aligned capillary stripes are a sign of active vascular remodeling. The rapidly following explosive increase in vascularization is not restricted to bundles of arteries appearing in the anteromedian sulcus. The mass of newly formed blood vessels are large immature peripheral arteries focused on the area of motoneurons and characterized by tortuous, occasionally bizarre courses (Fig. 12d,e). To our knowledge this course of events has neither been described nor illustrated in the literature.

With respect to periods of major increase in spinal cord vascularization, two studies should be mentioned [22, 33]. Both deal with investigations of rabbits and presented conflicting results, partly due to different observation periods. Sturrock [33] used a method of quantitative histology and showed a second steady but significant increase in the ventral and dorsal gray matter in a gestational time interval that might correspond to our observations in cattle.

The subsequent intrinsic restructuration process implements central (centrifugal) supply in addition to the existing peripheral system. At 11 weeks we observed strongly increasing vascular density of the gray matter, which is now much higher than in the marginal zone (Fig. 15). At the end of our observation period abundant supply through peripheral arteries still exceeds the contribution of sulcal/central arteries. In the adult spinal cord, the coexistence of both a centrifugal and a centripetal system has already been described by Adamkiewicz [38, 39]. He called the arteries involved in central (centrifugal) supply “sulcal” and “sulco-commissural” arteries, thus differentiating the extrinsic and the further intrinsic course of these blood vessels. Kadyi [37] followed Ross [40] using the earlier English nomenclature “central arteries” for both the intrafissural and the intraparenchymal section of the vessel. The significance of these two systems of supply in the spinal cords of vertebrates seems to be highly variable. Sterzi [8] listed types of supply for different classes of vertebrates in which the purely centripetal or centrifugal type is exceptional and combinations frequent. In cattle and human spinal cords, the chief supply is the central type in the enlargements and the peripheral in the thoracic cord. This corresponds to the central location of the neurons in the cord and the different ratios of gray and white matter between its regions (Fig. 12g–i; [17, 18]).

The development of a paired anterior spinal artery into a single vessel has been a matter of extensive discussions. His [6] reported paired anterior spinal arteries in human embryos of 10.9–13.8 mm in length and an unpaired artery in an embryo of 18 mm. His sole explanation of how the transition should go was medial displacement and fusion of the two primitive arteries in a way similar to the fusion of the two primitive aortae. This view was shared by others [7] but doubts were raised since Kadyi [37] had pointed out that with a few exceptions sulcal/central arteries in the adult leave the anterior spinal artery singly to only one hemicord. This significant observation is not well compatible with a fusion process. Sterzi [8] therefore suggested a more complex procedure. Similar to Evans [9] he had observed that the longitudinal channels of a primitive paired ASA are only stronger parts of a rope ladder-like vascular network, connected across the midline by transverse anastomoses. According to his observations he postulated a process by which only the channel on one side persists. Filling of sulcal/central arteries on the regression side would remain possible through the persistence of transverse anastomoses. In this way the sulcal/central arteries could keep their lateral relationship. Evans [10; footnote 13] agreed with the amendment that replacement of the paired tracts by a large vascular plexus would be another realistic alternative for the development of an exactly median ASA.

At this point of the discussion it is important to remember that detailed but sporadic illustrations of Sterzi [8] and Evans [9, 10] were based on very young specimens smaller than 20–30 mm in length from cattle, sheep and pig. This means that our observations of cattle began at a gestational age shortly before those previous studies ended. Our observations include an 11-week cattle specimen of 130 mm VBL (Figs. 15 and 16) and it is this much later fetal period in which a final reconstruction from a paired to a single anterior spinal artery takes place. According to the study presented here, the primary single anterior tract (Fig. 12a–c) from the embryonic period will regress (Figs. 8 and 9) when increasing metabolic demands develop. Transiently, paired longitudinal tracts reappear (Fig. 11) and give origin to large sulcal/central arteries for a central supply of each hemicord. The arteries emerge together with a burst of vascularization from the secondary spinal meninges. In the course of this significant modification of the vascular architecture, restoration of a final unpaired ASA is necessary again (Figs. 14, 15 and 16).

Nonetheless, the observations and considerations of Sterzi [8] and Evans [10] concerning the development of an unpaired anterior spinal artery are useful and important. A striking example for a unilateral origin of the ASA are the residual channels which have developed as an unpaired ASA on different sides of the midline in the specimen demonstrated in Fig. 16a,b. Furthermore, observation of changing paramedian courses right or left of the midline are common findings in the adult (Fig. 17). Anterior spinal arteries coursing with a considerable lateral deviation are frequently encountered at the level of incoming feeders (Fig. 14c and 17). Supply by long sulcal/central arteries at such locations must be based on the pre-existence of a rather wide anastomotic net. It is comprehensible that an anterior spinal artery which emerges from a wide vascular plexus can easily replace or include the small paired anastomotic channels and still keep a midline position as proposed by Evans ([10]; Fig. 15). If formation of a single common trunk fails, reunion of the paired segment after a common parallel course may happen or the less developed blood vessel will finally terminate as a central artery (Fig. 14a and 16c,d and 17a). Regression of one of the paired anterior tracts may be incomplete in sections of the thoracic cord. The small residual unilateral trunk may persist below the much larger ASA as part of the anterior pial system and give rise to several sulcal/central arteries on this side (Fig. 14d). This high range of normal developmental variations are the reason for the high rate of spinal vascular abnormalities in the adult. The final ASA is designed as a longitudinal anastomotic trunk and is the sole feeder of the system of central supply but it is also an essential pial feeder for peripheral supply in the ventral two thirds of the spinal cord (Fig. 14c,d; [17, 18, 41]). Parallel to the development of the definite anterior spinal artery goes a process of desegmentation [23], which means a numerical reduction of segmental feeders needed to reinforce the longitudinal tracts on the medullary surface. These arteries are called anterior and posterior radiculomedullary arteries. Their connections with the longitudinal tracts mostly occur in a “hairpin” configured course (Fig. 6d and 14c and 17b). Our observation of such “hairpin” arrangements at thoracic levels in a specimen of 8 weeks GA must be considered as a transitional manifestation, notwithstanding the problem of whether these vessels are of arterial or venous type (Fig. 6). Anterior and posterior radiculomedullary blood vessels are apparent in the 11-week specimen (Fig. 15) but the posterior surface of the spinal cord in this specimen is still covered by a coarse mesh of unorganized blood vessels. Development of the final pattern of posterior spinal arteries (Fig. 1e) is reported to last until the 15th and 20th week of fetal life [4, 5]. The final configuration of the entire extrinsic supply pattern follows the general principles discussed above but in the end will exhibit an entirely individual design.

## Conclusion

Radiographic techniques including microangiography of injected specimens are very useful in investigations dealing with vascularization processes of tissues and organs in the course of prenatal development. The observation period for the spinal cord should not be restricted to the embryonic period. Otherwise, complex or repeated reconfigurations of the vascular supply pattern induced by histogenesis and changing metabolic demands may be missed. The development of the anterior fissure and the secondary meninges which generate a burst of vascularization, play a key role in the implementation of a central system of arterial supply which is predominant in the enlargements of the spinal cord in higher mammals.
